# Coarse-Grained Molecular Dynamics of pH-Sensitive Lipids

**DOI:** 10.3390/ijms24054632

**Published:** 2023-02-27

**Authors:** Isabel Lado-Touriño, Arisbel Cerpa-Naranjo

**Affiliations:** Engineering Department, School of Architecture, Engineering and Design, Universidad Europea de Madrid, 28670 Villaviciosa de Odón, Spain

**Keywords:** pH-sensitive lipids, coarse-grained, molecular dynamics

## Abstract

pH-sensitive lipids represent a class of lipids that can be protonated and destabilized in acidic environments, as they become positively charged in response to low-pH conditions. They can be incorporated into lipidic nanoparticles such as liposomes, which are able to change their properties and allow specific drug delivery at the acidic conditions encountered in some pathological microenvironments. In this work, we used coarse-grained molecular-dynamic simulations to study the stability of neutral and charged lipid bilayers containing POPC (1-palmitoyl-2-oleoyl-sn-glycero-3-phosphocholine) and various kinds of ISUCA ((F)2-(imidazol-1-yl)succinic acid)-derived lipids, which can act as pH-sensitive molecules. In order to explore such systems, we used a MARTINI-derived forcefield, previously parameterized using all-atom simulation results. We calculated the average area per lipid, the second-rank order parameter and the lipid diffusion coefficient of both lipid bilayers made of pure components and mixtures of lipids in different proportions, under neutral or acidic conditions. The results show that the use of ISUCA-derived lipids disturbs the lipid bilayer structure, with the effect being particularly marked under acidic conditions. Although more-in depth studies on these systems must be carried out, these initial results are encouraging and the lipids designed in this research could be a good basis for developing new pH-sensitive liposomes.

## 1. Introduction

Nanoparticles that are sensitive to a variety of physical and chemical stimuli have potentially important applications in drug delivery, and currently their development is an area of intensive research [1,2,3]. Although there are many different kinds of stimuli-sensitive nanoparticles, such as those based on polymers, colloids or liquid crystals, it is worth highlighting among them liposomes, spherical-shaped vesicles that consist mainly of phospholipids forming a lipid bilayer surrounding an aqueous core [4]. They show many advantages over other types of nanostructures, such as easy production methods, good control of liposome size and ease of charge with high drug/lipid ratios. They are usually made of lipids that mimic biological membranes [5]. Thus, they can be used as simple models to understand and characterize real cell membranes, which are sometimes difficult to study. Different activation methods have been used to deliver drugs contained inside stimuli-sensitive liposomes in a controlled manner, such as light, strain, pH, heat or magnetic and electric fields, among others [6,7,8,9,10,11,12]. Specifically, pH-sensitive liposomes are able to respond to pH changes. Their structures contain functional groups that protonate/deprotonate as a function of pH value, giving rise to morphological changes in their lipid bilayers. Thus, alteration in pH (as in tumor tissues, which present low pH due to an increased glycolysis, which stimulates the production of lactic acid [13]), can cause release of the entrapped drug, due to instability of the bilayers when changing their structure [14,15,16,17]. This pH change in cancer cells is a key factor for the use of these liposomes in cancer treatment.

Liposomes are usually formulated using modified natural diacyl-chain phospholipid components, such as phosphatidylethanolamine (PE), phosphatidylcholine (PC), phosphatidylserine (PS) or oleic acid (OA) [18], among others. The phospholipid membrane, which forms the lipid bilayer of a liposomal structure, consists of two clearly differentiated parts: a hydrophobic tail and a hydrophilic head. Although there are numerous chemical structures that can act as pH-sensitive hydrophilic headgroups, in this work, imidazole-derived molecules were chosen for the design of new pH-sensitive lipids, as they can protonate even in response to weakly acidic pH [19,20]. Specifically, Provent et al. [21] synthesized one of such molecules, ISUCA ((F)2-(imidazol-1-yl)succinic acid) and used this new imidazole derivative as a probe agent to measure the extracellular pH in C6 cell gliomas in rat brains. Due to its excellent response to pH changes, we selected the ISUCA molecule to build the headgroups of our new pH-sensitive lipids.

Among the different methods that are successfully applied to the study of lipid bilayers and liposomes, notable are those based on computational approaches. Thus, all-atoms simulations have been employed for many years to study and understand a wide variety of properties related to these systems [21,22,23,24]. However, this kind of method is very compute-intensive and its use is limited to small systems. To study large-scale structures or processes that need very long time scales to complete, using other type of simulation approaches is mandatory. Coarse-grained (CG) methods seems to be a good alternative. They involve grouping together several atoms into single sites, the so-called beads, and significantly reduce the number of interactions between particles that need to be calculated and hence, computational cost. Thus, many properties of interest in mesoscopic-scale systems can be studied using CG simulations [25,26,27]. In conclusion, CG models allow for studies of larger systems for longer times, compared with all-atom models. They are fast enough to simulate processes occurring even at a nanosecond time scale. For all these reasons, the CG approach has experienced huge success in recent years. Several different CG models have been developed and applied to the study of diverse atomistic systems [28,29,30,31]. Notable among these is the MARTINI forcefield [28]. This forcefield has been successfully used to study different biological systems such as lipids [32], liposomes [33], proteins [34], carbohydrates [35] and amino acids [36]. 

In this work, we report novel types of lipids derived from the ISUCA molecule and use them to build models of pure-lipid bilayers (ISUCA and POPC-(1-palmitoyl-2-oleoyl-sn-glycero-3-phosphocholine)), as well as mixed-lipid bilayers made of POPC and ISUCA-derived lipids. We studied these new bilayers under neutral and acidic conditions, using CG molecular dynamics (CGMD).We explored their structural stability by means of calculation of some characteristic parameters of the lipids, such as the average area per lipid, hydrophobic thickness, second-rank order parameters and diffusion coefficients. Bearing in mind that the ultimate goal of our research is the design and synthesis of new pH-sensitive liposomes made of this kind of lipid, we think that we can start our study through the simulation of the structural and dynamical properties, under different pH-conditions, of smaller and simpler models, such as those presented herein. If the results are satisfactory, the best structures may be subsequently used to produce liposomes in the laboratory.

## 2. Results and Discussion

The characterization of the lipid bilayers was carried out by computing structural and dynamical properties from the CGMD trajectory obtained from the simulations. The results for both kind of properties are presented separately in the following sections:

### 2.1. Structural Properties: Equlilibrium Area per Lipid, Hydrophobic Thickness and Second-Rank Order Parameter

The equilibrium area per lipid (APL) and the hydrophobic thickness of all lipid bilayers considered in this study, are shown in Table 1. The composition of each system is described in the Section 3, as detailed below. Both properties provide information about the structure of the lipid bilayer. If, for some reason, this structure changes, this should be reflected in the values of APL and hydrophobic thickness.

**Figure 1:**
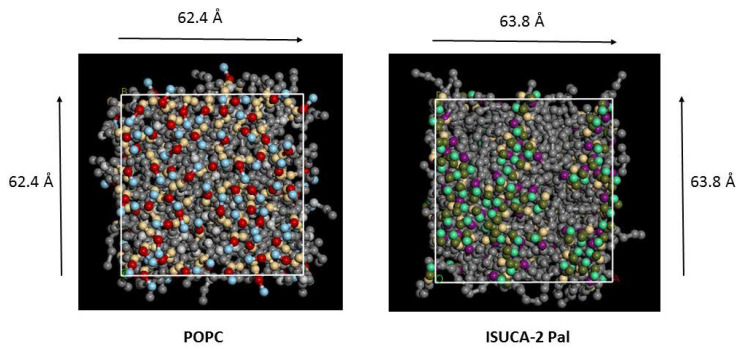
Top view of pure POPC and ISUCA-2 Pal lipid bilayers. Grey balls represent hydrocarbon tails.

The APL is defined as the area of the surface bilayer (perpendicular to the bilayer) divided by the number of lipids in each monolayer. For instance, the value for POPC (67 Å^2^) is obtained by multiplying the dimensions of its surface (see Figure 1: 62.4 Å × 62.4 Å) by the number of lipids in the monolayer (58). It is an important property of biological and synthetic membranes. In more ordered systems, the lipids are more densely packed, which results in smaller distances between them and lower APL values. Thus, it is lower for more ordered systems and also for lipids with smaller headgroups [37,38]. The APL of POPC calculated in this work, is close to the experimental value of 68 Å^2^ at high hydration levels [39,40]. The number of water molecules per lipid is 63.4 in our models, which means an elevated hydration level. To rule out the effect of lipid size on the APL (as smaller lipids usually have lower APL values), the headgroup volume was calculated for both kinds of lipids (POPC and ISUCA-derived lipids), and is shown in the last two rows of Table 1. Hydrophobic tails are not taken into account, as they are the same for all lipids, that is to say, what differentiates POPC from ISUCA-derived lipids is only the headgroup. Although the ISUCA headgroup is smaller, the APL of ISUCA-derived lipids is larger, which could indicate less-structured bilayers. The increase in APL values is observed in both pure and mixed bilayers, although it is much more accentuated in pure systems (see first four rows in Table 1). Mixed bilayers present APL values falling between those of the pure bilayers. Furthermore, the effect of introducing an unsaturated fatty acid in the lipid tails, results in an increase in APL values. The ISUCA-2 Ol bilayer, containing two unsaturated chains in its structure, present the highest value of all (73.1 Å^2^). This result is well-known and has been found by many other authors [41,42,43,44] in different lipid bilayers, as unsaturated chains give rise to less-ordered structures, which in turn leads to higher APL values. All mixed bilayers present APL values lower than that of pure POPC. This could indicate that, when ISUCA-derived lipids are introduced into a POPC membrane, its structure is somewhat disrupted and its disorder increases. Protonation of the structure leads to a slight increase in APL.

**Figure 2:**
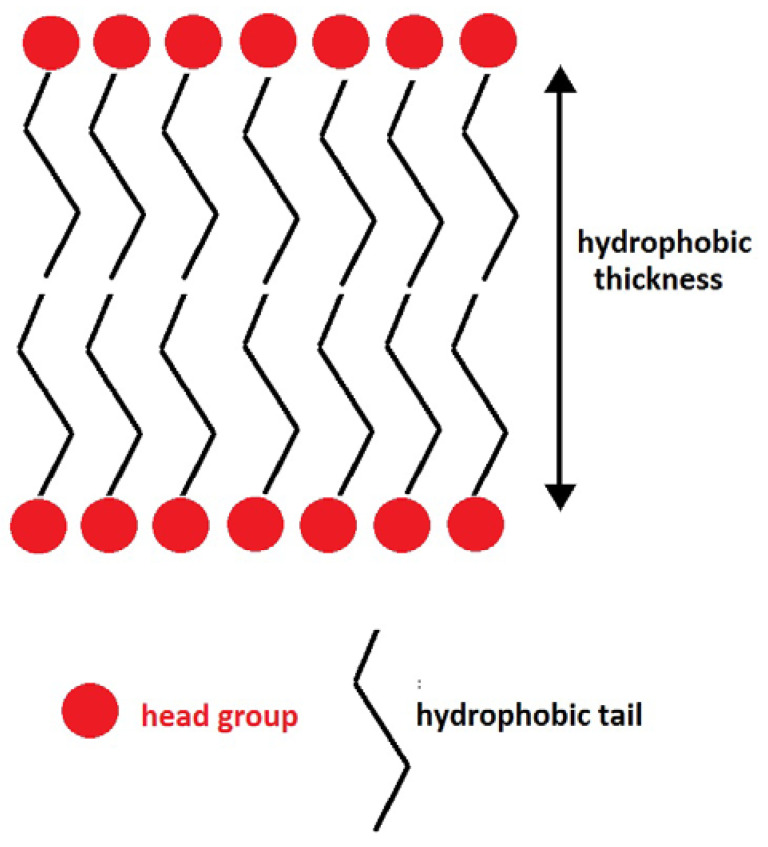
Schematic representation of a lipid bilayer and hydrophobic thickness.

The hydrophobic thickness, the thickness of the hydrocarbon tails, follows the same trend as that of the APL values. For clarity, Figure 2 shows a schematic representation of a lipid bilayer, indicating how hydrophobic thickness is defined. It is well known that ordered structures present higher hydrophobic-thickness values, as they are more rigid and longer [39,41]. Unsaturated chains decrease hydrophobic thickness. In general, bilayers containing ISUCA-derived lipids, both pure or mixed, present lower hydrophobic thicknesses. The only exception is ISUCA-2 Pal (see second row of Table 1). This could be because it is the only lipid in this study with two saturated hydrocarbon chains in its structure, which are longer than unsaturated chains. The effect of concentration and protonation on the hydrophobic thickness is clearly seen in the last three rows of the table (only POPC/ISUCA-Pal-Ol bilayers were considered for this study). Increasing the ISUCA-derived lipid concentration (pure POPC: 14.8 Å; 90% POPC: 13.1 Å; 50% POPC: 12.9 Å) or protonating the imidazole ring (12.5 Å, compare the last two rows of Table 1 for lipid bilayers containing 90% POPC) shortens the hydrophobic thickness, which points, once again, to more disordered structures. However, hydrophobic-thickness results must be taken with caution, as the calculated standard deviations of the data are high.

As an example, Figure 1 shows the top view (normal for the lipid bilayer) of two of the bilayers studied (pure POPC and ISUCA-2 Pal) at the end of the simulation. For clarity, water molecules, located above and below the lipid bilayer, are hidden. As can be seen, the pure POPC bilayer shows a more regular and ordered distribution of lipids, in accordance with APL and hydrophobic-thickness results. ISUCA-derived lipids heads tend to agglomerate, resulting in more disorganized structures.

The second-rank order parameter, P_2_, is another way of quantifying the packing and ordering of lipids. It represents a measure of the alignment of hydrocarbon tails with the bilayer normal.

P_2_ is defined as

**Figure 3:**
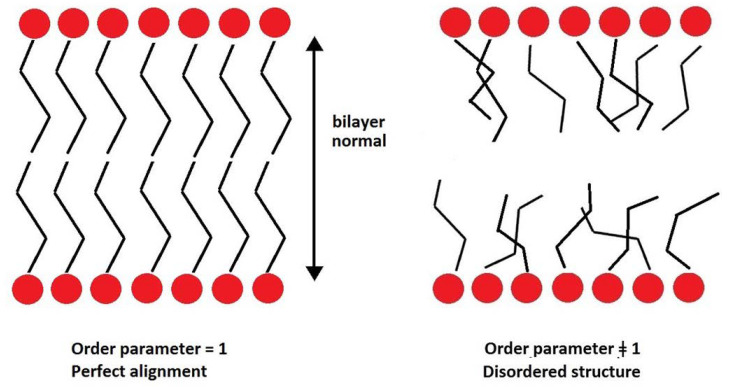
Relationship between P_2_ and degree of order of a lipid bilayer.

**Figure 4:**
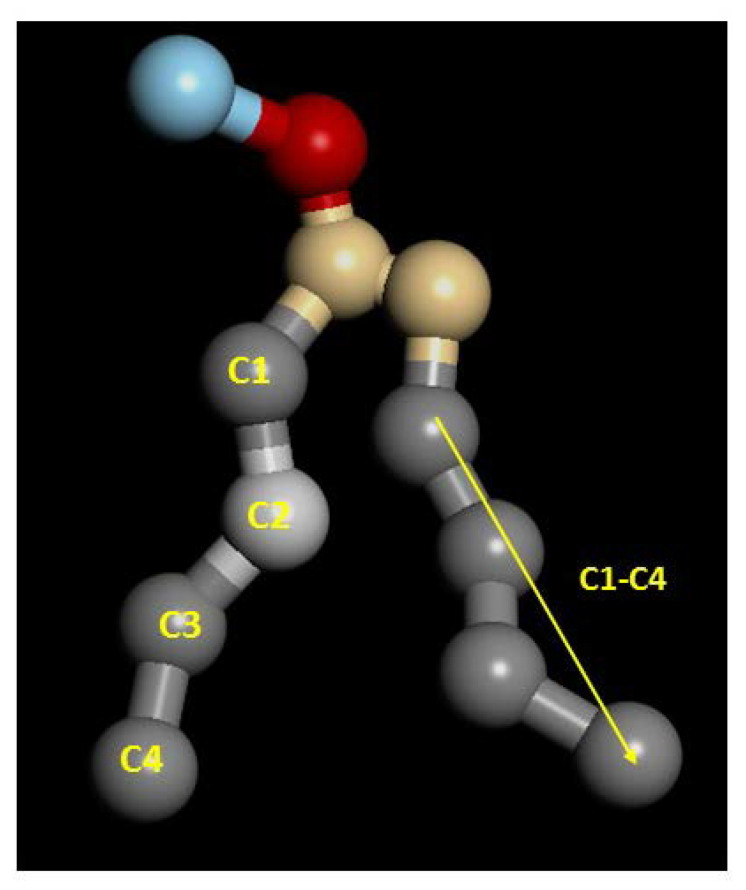
Numbering of beads used to calculate order parameters.

P_2_ = <1/2 (3cos2θ − 1)> (1)

with θ the angle between the direction of a bond (or a vector) and bilayer normal. Perfect alignment of bonds of the hydrocarbon tails with the lipid bilayer is indicated by P_2_ = 1, and a random orientation with P_2_ = 0 (Figure 3). In this work, two different P_2_ parameters were defined and calculated: the bond order parameter (P_2b_), for consecutive tail bonds, and the tail order parameter (P_2t_), for the vector joining beads C1 and C4 of the hydrocarbon tail (see Figure 4).

**Figure 5:**
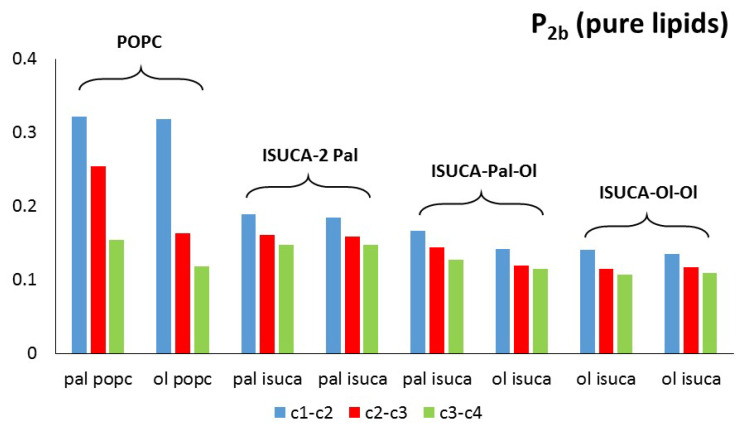
P_2b_ for pure lipids: pal = palmitic hydrocarbon chain, ol = oleic hydrocarbon chain.

**Figure 6:**
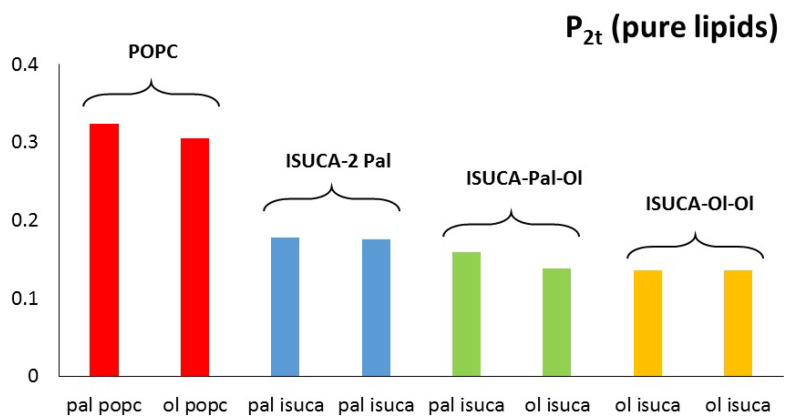
P_2t_ for pure lipids: pal = palmitic hydrocarbon chain, ol = oleic hydrocarbon chain.

Figure 5 and Figure 6 show the calculated values of both order parameters, P_2b_ and P_2t_, of the pure bilayers considered in this study. It must be noted that the results of each individual hydrocarbon chain (palmitic- and oleic-acid-derived chains) are presented separately for all lipids.

It is well-known, and also found in this work, that the order parameter decreases with the distance from the headgroup (c1-c2 > c2-c3 > c3-c4), with the bonds closest to the hydrophilic head being, more ordered than the bonds farther apart. The POPC P_2b_ values of these bonds are around 0.3. These results agree with typical experimental values found in reference [45]. The calculated average P_2t_ for POPC (average value for the two hydrophobic chains) has a value of 0.31, which is close to the result found by other authors [46]. In the same way, P_2b_ parameters are similar to those calculated in reference [47]. Moreover, the P_2_ of the saturated hydrocarbon chains (pal) are greater than those of the unsaturated chains (ol), which indicates a more regular packing of the former. This result applies to both P_2b_ and P_2t_. In addition, in all systems studied in this work, the ISUCA-derived lipids have a lower degree of order, as evidenced by their smaller P_2_ values (note the decreasing trend of the P_2_ values from left to right in Figure 5 and Figure 6). As expected, the unsaturated chains show smaller P_2_ values.

**Figure 7:**
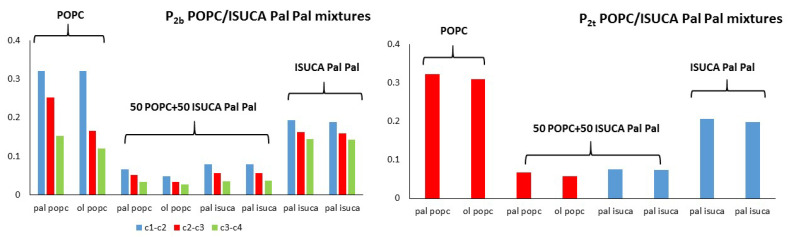
P_2t_ and P_2b_ for POPC/ISUCA-Pal Pal mixtures.

**Figure 8:**
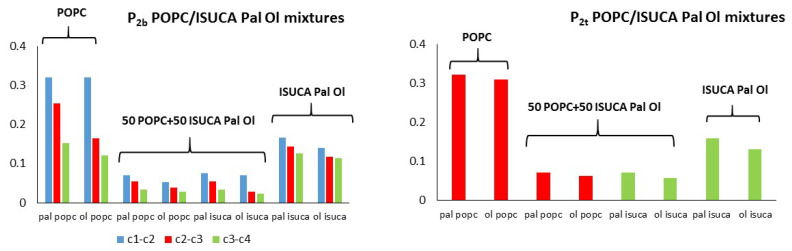
P_2t_ and P_2b_ for POPC/ISUCA-Pal Ol mixtures.

**Figure 9:**
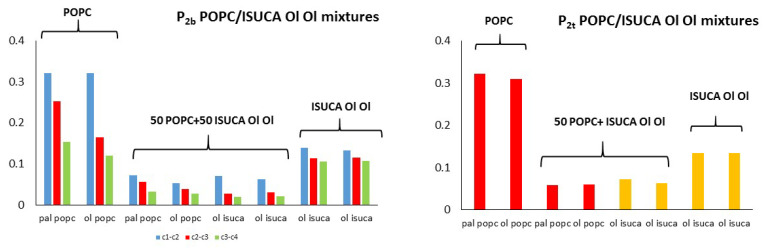
P_2t_ and P_2b_ for POPC/ISUCA-Ol Ol mixtures.

Figure 7, Figure 8 and Figure 9 depict results for 50:50 lipid mixtures. To enable a comparison between pure and mixed bilayers, P_2_ values of pure lipids are also shown in these figures.

POPC/ISUCA mixtures follow the same trend as that of bilayers made of pure lipids: a smaller-order parameter for bonds farther away from headgroups (c1-c2 > c2-c3 > c3-c4), and unsaturated chains. The most remarkable result shown in these figures is the fact that that both P_2t_ and P_2b_ parameters of pure-lipid bilayers are clearly larger than those of their respective mixtures. This clearly indicate that mixed bilayers are less ordered than pure bilayers, as previously found by APL and hydrophobic-thickness calculations. 

**Figure 10:**
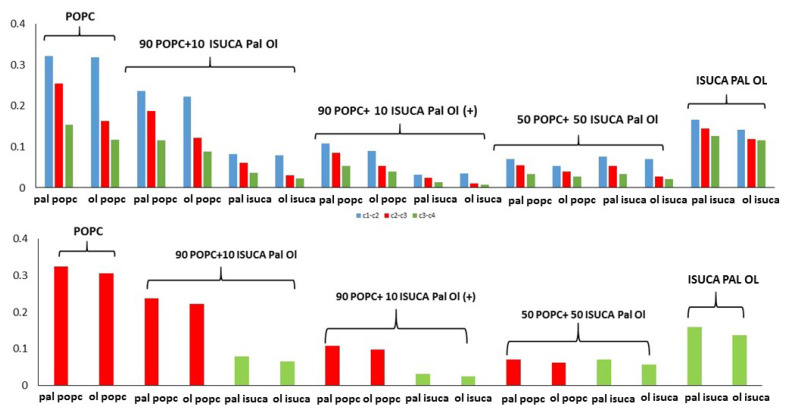
P_2t_ (**top**) and P_2b_ (**bottom**) for POPC/ISUCA Pal Ol mixtures of varying composition.

Finally, the effect of lipid-bilayer composition, as well as that of protonation of the ISUCA moiety in POPC/ISUCA-Pal Ol mixtures, is shown in Figure 10.

As expected, both P_2t_ and P_2b_ parameters decrease when the ISUCA-derived lipid concentration increases (0% > 10% > 50%). What is worth noting is the effect of protonation (marked with a plus sign in Figure 10). A noticeable reduction in both the P_2t_ and P_2b_ parameters is observed (compare values for protonated and unprotonated 90 POPC + 10 ISUCA Pal Ol bilayers), which indicates a less-ordered structure in the protonated case. This result is very promising in terms of accomplishing our goals, the design of new pH-sensitive liposomes, as it shows that a pH change could lead to a variation in the lipid bilayer structure, towards a more disordered state. 

In short, the introduction and protonation of ISUCA-derived lipids in a POPC bilayer causes a significant increase in disorder, as indicated by the values of the three structural parameters, APL, hydrophobic thickness and P_2_, calculated in this work.

### 2.2. Dynamic Properties: Diffusion Coefficient

In addition to characterizing the structural parameters of these new membranes, the dynamics of the lipids was also studied by calculating the lipid lateral-diffusion coefficient, D. D can also provide information about the structure and ordering of the membrane. It is related to lipid mobility inside each monolayer. Thus, lipid lateral diffusion reflects the translational motion of lipids along the monolayer. Highly fluctuating and mobile lipids are indicative of disorder. The packing of lipids and how they are arranged in the bilayer are important properties that seem to strongly affect the lateral-diffusion coefficients [48]. In general, a closer packing of the lipids and well-ordered bilayers results in a decrease in D and reduced lateral diffusivity.

D was derived from the slope of the mean squared displacement (MSD) of lipids versus time plots, assuming Fickian diffusion:

(2) $$MSD=\overset{N}{\underset{i=1}{\sum }}{\langle \left(r_{i}\left(t+\Delta t\right)-ri\left(t\right)\right)}^{2}\rangle =4\cdot D\cdot t$$

where vector *r_i_*(*t*) is the position of the *i*th bead at time *t*, and *r_i_*(*t* + Δ*t*) the position of the same bead, an interval Δ*t* later.

The calculated *D* values are shown in Table 2.

The calculated lateral D of all lipid bilayers are of the order of 10^−7^ cm^2^·s^−1^, typical for lateral-diffusion rates of lipids in the fluid phase [49]. The POPC diffusion coefficient is close to the experimental result [50]. The diffusion coefficients of pure systems are larger for ISUCA-derived lipid bilayers (compare the first four rows of Table 2) and a noticeable increase is seen when unsaturated chains are introduced into the lipid composition (2 Pal: 3.3; Pal Ol: 5.6; Ol Ol: 5.7). The ISUCA-2 Pal system present a diffusion coefficient that is larger than that of pure POPC (2.2), but lower than those of the other two mixed bilayers. These results are in accordance with results found in previous investigations [46], which, moreover, established a relationship between APL and D. Apajalahti et al. [51] also found a clear positive correlation between D values and disordered structures: the more ordered the system is, the slower the diffusion. Once again, our results seem to indicate that ISUCA and oleic acid help to disrupt lipid bilayers, making them more fluid, disordered and dynamic.

Mixed bilayers present D values falling between those of pure bilayers. The greater the amount of ISUCA, the greater the D value (pure POPC: 2.2; 90% POPC: 2.4; 50% POPC: 3.4): the protonated structure shows a slightly higher D value (2.6) than that of the unprotonated system (2.4), although the effect of protonation and hydrocarbon-chain composition on D is less accentuated in mixed systems than in pure systems.

Although lipid bilayers are not the ultimate goal of our research, we think that the use of such simpler models can be useful to study the behavior of more complex systems, such as liposomes, under different pH conditions. We have found that by using ISUCA-derived headgroups, changing the composition of the hydrocarbon tails and lowering the pH, all parameters calculated (APL, P_2_, D, and hydrophobic thickness) point to an increase in disorder in the lipid bilayers. This, although not definitive, could be a good starting point to design new pH-sensitive liposomes made of this kind of lipids. Future work must focus on varying the proportions of the three ISUCA derivatives, different degrees of protonation and, of course, the CGMD of liposomes, incorporating the most promising components found in our lipid-bilayer simulations.

## 3. Materials and Methods

The models used to represent the lipid bilayers, as well as the computational procedure followed to calculate the properties discussed in the previous section, are presented below:

### 3.1. Models

**Figure 11:**
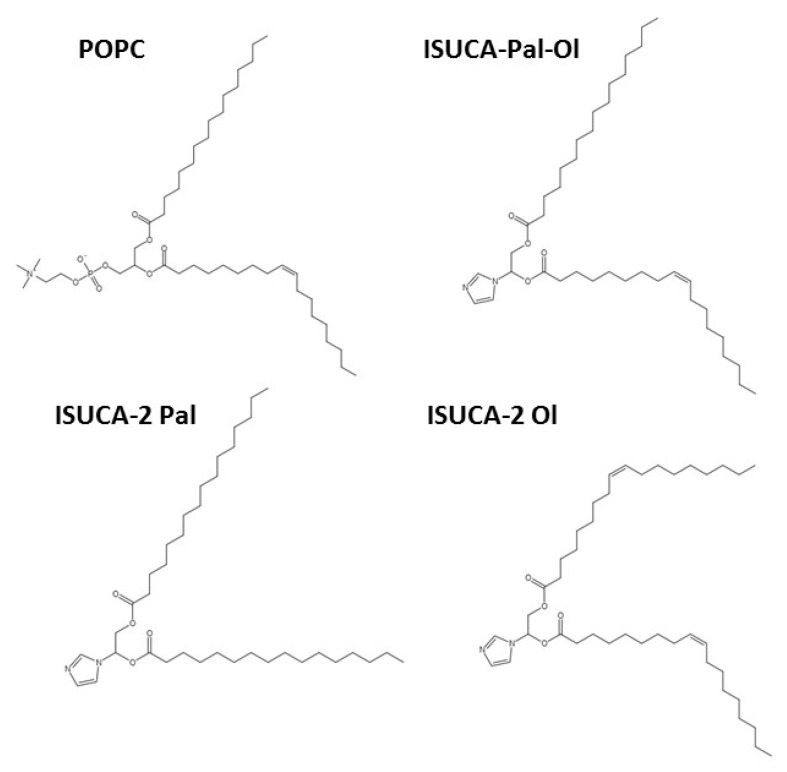
Chemical structures of POPC and ISUCA-derived lipids used in the construction of the lipid bilayers.

The chemical structures used to construct the lipid bilayers are shown in Figure 9. We designed three different ISUCA-derived lipids: they all consisted of a hydrophilic head (ISUCA) and two hydrophobic tails made of hydrocarbon chains derived from palmitic acid (ISUCA-2 Pal), oleic acid (ISUCA-2 Ol) or both (ISUCA-Pal-Ol). The POPC structure is also shown in Figure 11.

Different lipid bilayers were then built from lipids shown in Figure 11: pure lipid bilayers (POPC and ISUCA-derived lipids), as well as their mixtures in different proportions. Although we are mainly interested in mixed systems, pure bilayers were used in this study for the sake of comparability. The composition of all models considered in this study, is shown in Table 3. To study the effect of concentration and protonation, only ISUCA-Pal Ol structures were used.

**Figure 12:**
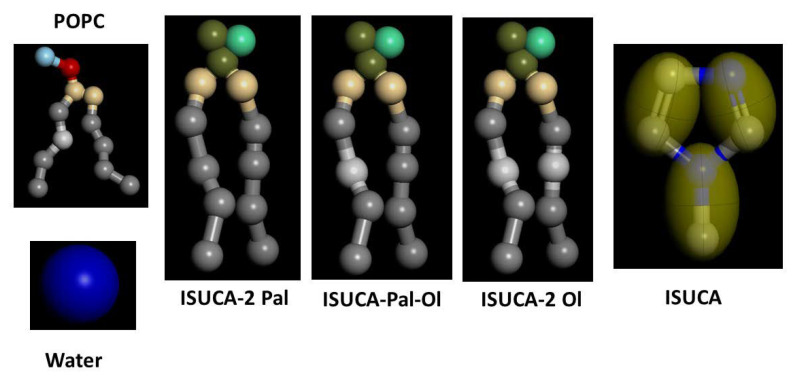
Structures of the molecules in the CG model.

The lipids were represented by beads, as shown in Figure 12. This bead representation is typical of the MARTINI forcefield and is well documented in reference [28]. The Martini forcefield was chosen to calculate bead interactions, and has been successfully applied to the study of numerous systems, including both liposomes [33] and lipid bilayers [34]. For POPC, the headgroup consisted of two hydrophilic beads to represent the amine group and the phosphate moiety, and two intermediately hydrophilic ones for the glycerol moiety. Each of the two tails was modeled by four hydrophobic particles. We took the parameter set for POPC from ref [28]. The tails of the ISUCA-derived lipids were represented in the same way, while the headgroup consisted of three beads for the imidazole group and two beads for glycerol (right panel in Figure 10). Because this structure was not previously parameterized in the Martini forcefield and because of the lack of experimental data, bonded parameters for the headgroups of ISUCA-derived lipids were obtained from all-atoms molecular dynamics. The force-field parametrization procedure as well as the parameters obtained from the all-atoms calculations that were subsequently used in our model, are given in the Supplementary Material. The coarse-grained model used to parameterize the ISUCA headgroup and the atomistic model of the lipid bilayer are shown in Figure S1 and Figure S2, respectively. Figure S3 depicts a comparison of structural parameters obtained from both atomistic and CG molecular dynamics simulations. The functional forms and parameters used to describe bonded interactions of the headgroup of ISUCA-derived lipids are listed in Table S1. Some other parameters for the imidazole group were taken from reference [29]. The solvent was modeled by individual hydrophilic particles, each representing four real water molecules. A specific problem of the CG model used (and which we found in our simulations) is that the water has a freezing temperature that is somewhat too high, compared to real water. This phenomenon is accentuated in systems containing nucleation sites, confined geometries and periodic conditions, as in our models. This results in the solidification of water and leads to unrealistic results. To overcome the effects of higher freezing temperatures, we used 30% antifreeze particles in our calculations, as recommended in reference [28].

All lipid bilayers contained 116 lipids (58 in each monolayer) and 1840 water beads (including antifreeze particles). The composition of each bilayer, as well as the model dimensions used in the simulations, are shown in Table 4.

### 3.2. Calculation Method

All systems were simulated with the Mesocite module [52] of the Materials Studio 7.0 software [53], using periodic boundary conditions (cell parameters are shown in Table 4) in the NPT ensemble (number of particles N, pressure P and temperature T, constant) and an effective simulation time of 4 μs using a time step of 20 fs. The effective simulation time is defined as the actual simulation time multiplied by a factor of 4. This scaling factor was previously found to reproduce both lipid lateral-diffusion rates and the self-diffusion of water [54]. Monitoring the APL value indicated that the systems had reached equilibrium after 4 μs. The temperature was controlled by a Nose thermostat [55] and kept constant at 310K. An Andersen barostat [56] was used to maintain pressure at 1 bar. To calculate Coulomb interactions between charged particles, the Ewald summation method was used and a bead-based cutoff method was applied for van der Waals interactions. The cutoff distance for both interactions was 12.5 Å. Due to the long computational times required to characterize the systems, all simulations started in the bilayer state. Before starting the CGMD simulations, the starting configurations of the lipid bilayers were energy minimized to remove any possible bad contacts among the atoms.

## 4. Conclusions

In this work, we calculated the structural and dynamic properties of new lipid bilayers made of POPC and/or ISUCA-derived lipids, using CGMD simulations. We think that these new chemical structures can be used as a good starting point to study and, if the results are promising, synthesize pH-sensitives liposomes made of the lipids studied in this work. The values obtained for the area per lipid, hydrophobic thickness, order parameter and diffusion coefficients seem to indicate an increase in disorder of the structures when ISUCA-derived lipids form part of the bilayer. The area per lipid increases with increasing concentration of ISUCA-derived lipids and the introduction of unsaturated bonds in the hydrocarbon tails. The increase in area per lipid is observed in both pure and mixed bilayers. The same trends are observed for order parameters. They are smaller when greater amounts of unsaturated ISUCA-derived lipids are present in the structure. Hydrophobic thickness points in the same direction, although due to higher standard deviations of the data, these results should be interpreted with caution. Diffusion coefficients are larger for ISUCA-derived lipid bilayers and a noticeable increase is seen when unsaturated chains are introduced into the lipid composition. Protonating the ISUCA-derived headgroup probably disrupts the bilayer, as indicated by a marked decrease in the order parameter compared to the non-protonated structure, a slight increase in the diffusion coefficient and hydrophobic thickness, and a larger APL value.

Although more calculations of more diverse structures, both lipid bilayers and liposomes, with varying concentrations of ISUCA-derived lipids and different degrees of protonation are required to reach firmer conclusions, we think that these initial results are encouraging. The introduction of ISUCA disrupts the structure of the POPC bilayer, and therefore the new lipids designed in this research could be a good basis for developing new pH-sensitive liposomes.

## Figures and Tables

**Table 1:** APL and hydrophobic thickness of bilayers. The calculated volumes of the lipid headgroups are also shown in the last two rows.

Model	APL (Å^2^)	Hydrophobic Thickness (Å)
POPC	67.0 ± 0.1 (exp: 62–68) ^1^	14.8 ± 1.2
ISUCA-2 Pal	70.3 ± 0.2	15.7 ± 0.4
ISUCA-2 Ol	73.1 ± 0.1	14.4 ± 0.7
ISUCA-Pal Ol	71.3 ± 0.1	14.5 ± 0.5
50:50 POPC/ISUCA-2 Pal	70.3 ± 0.1	14.8 ± 0.6
50:50 POPC/ISUCA-2 Ol	70.4 ± 0.1	11.7 ± 0.4
50:50 POPC/ISUCA-Pal-Ol	70.4 ± 0.2	12.9 ± 0.8
90:10 POPC/ISUCA ISUCA-Pal-Ol	70.4 ± 0.1	13.1 ± 0.5
90:10 POPC/ISUCA+-Pal-Ol (protonated ISUCA)	70.8 ± 0.1	12.5 ± 0.5
**Headgroup**	**Volume (Å^3^)**	
POPC	170	
ISUCA	158	

^1^ Depending on the hydration level of the bilayer. Interaction of head groups with surrounding water influences APL.

**Table 2:** Calculated *D* of the different bilayers studied.

Model	*D* (×0^7^ cm^2^·s^−1^)
POPC	2.2 (exp.: 1.9)
ISUCA-2 Pal	3.3
ISUCA-2 Ol	5.7
ISUCA-Pal Ol	5.6
50:50 POPC/ISUCA-2 Pal	3.0
50:50 POPC/ISUCA-2 Ol	3.4
50:50 POPC/ISUCA-Pal-Ol	3.4
90:10 POPC/ISUCA ISUCA-Pal-Ol	2.4
90:10 POPC/ISUCA+-Pal-Ol	2.6
(protonated ISUCA)	

**Table 3:** Composition of the bilayers studied by CGMD.

Pure Lipids	Mixtures
POPC	50:50 POPC/ISUCA-2 Pal
ISUCA-2 Pal	50:50 POPC/ISUCA-2 Ol
ISUCA-2 Ol	50:50 POPC/ISUCA-Pal-Ol
ISUCA-Pal Ol	90:10 POPC/ISUCA-Pal-Ol
	90:10 POPC/ISUCA^+^-Pal-Ol (protonated ISUCA)

**Table 4:** Number of beads and cell dimensions of the bilayers studied by CGMD.

Model	No. of POPC	No. of ISUCA	Model Dimensions (Å^3^)
POPC	116		
ISUCA-2 Pal	0	116	63.8 × 63.9 × 90.0
ISUCA-2 Ol	0	116	65.1 × 65.1 × 87.9
ISUCA.-Pal Ol	0	116	64.3 × 64.3 × 89.5
50:50 POPC/ISUCA-2 Pal	58	58	64.3 × 63.4 × 91.9
50:50 POPC/ISUCA-2 Ol	58	58	64.3 × 63.4 × 91.9
50:50 POPC/ISUCA-Pal-Ol	58	58	64.3 × 63.4 × 91.9
90:10 POPC/ISUCA ISUCA-Pal-Ol	104	12	64.3 × 63.5 × 92.0
90:10 POPC/ISUCA+-Pal-Ol (protonated ISUCA) ^1^	104	12	64.4 × 63.5 × 91.9

^1^ Cl^−^ counterions were added to ensure the overall neutrality of the system under acidic conditions. They were represented by a charged Qai particle.

## Data Availability

Not applicable.

## Supplementary Materials

The following supporting information can be downloaded at https://www.mdpi.com/article/10.3390/ijms24054632/s1. References [29,57,58,59,60,61,62] are cited in the supplementary materials.
