# Supplementary material for: Coarse-Grained Molecular Dynamics of pH-Sensitive Lipids
Source: Int J Mol Sci. 2023 Feb 27;24(5):4632. doi: 10.3390/ijms24054632 (PMC10003205; doi:10.3390/ijms24054632)
Supplement: Supplementary file 1 [file ijms-24-04632-s001.zip › Supplementary material.pdf]

Supplementary material to

Coarse-grained molecular dynamics of pH-sensitive lipids

Isabel Lado Touriño [1,*], Arisbel Cerpa-Naranjo[2]

[1] Engineering Department, School of Architecture, Engineering and Design, Universidad Europea de Madrid, Villaviciosa de Odón, Spain
[2] Engineering Department, School of Architecture, Engineering and Design, Universidad Europea de Madrid, Villaviciosa de Odón, Spain

* Correspondence: misabel.lado@universidadeuropea.es

Molecular Dynamics

Molecular dynamics (MD) is a computer simulation method that predicts how every atom in a molecular system will move in space during a certain period of time (the so-called simulation time) [1]. If the positions of all atoms in a system are known, the force exerted on each atom by all other atoms can be calculated, and classical Newtonian dynamic equations can be used to predict the spatial position of each atom as a function of time, thus creating the so-called MD trajectory. A wide variety of important processes such as conformational changes, transport across membranes or ligand docking, among others, can be studied by analyzing a MD trajectory.

The forces in a MD simulation are calculated from a set of equations and parameters known as forcefield [2]. More precisely, a forcefield is used to calculate the potential energy of a system of atoms as a function of of intramolecular (for instance, energies associated with covalent bonds or angles between bonds) and intermolecular (electrostatic and van der Waals energies) energy components. Once the potential energy is known, the force acting upon an atom is determined by the gradient of the potential energy, with respect to atomic displacements.

Molecular systems can be modeled at different levels of spatial resolution, which results in two different types of MD simulations. In atomistic models, all atoms are explicitly simulated. In coarse-grained models, the system is represented by a reduced number of degrees of freedom. This is done by grouping several atoms into a single interacting site called bead (see the coarse-grained model of the imizadole ring in figure S1. The transparent yellow ellipses are the beads that represent this molecule). Due to the reduction in the degrees of freedom, coarse-grained simulations require less computation resources and enable extended timescales and larger systems to be addressed.

Coarse-Grained Model Building and parameterization

The parameters of the forcefield need to be known to calculate the potential energy of the system. Most parameters used in this work are well known and can be found in references [3, 4] However, some of the interactions of the new lipids has to be parameterized. The coarse-grained model of the ISUCA-derived headgroup is shown in Figure S1. It consisted of two SP1 beads and one SC4 bead to represent the N containing groups and the C=C, respectively. The glycerol moiety, as well as the hydrocarbon chains were represented by the same model as that

used to described POPC in reference [3]. For the charged version, the top-left SP1 bead was replaced with a charged SQd particle following the model for charged histidine [4].

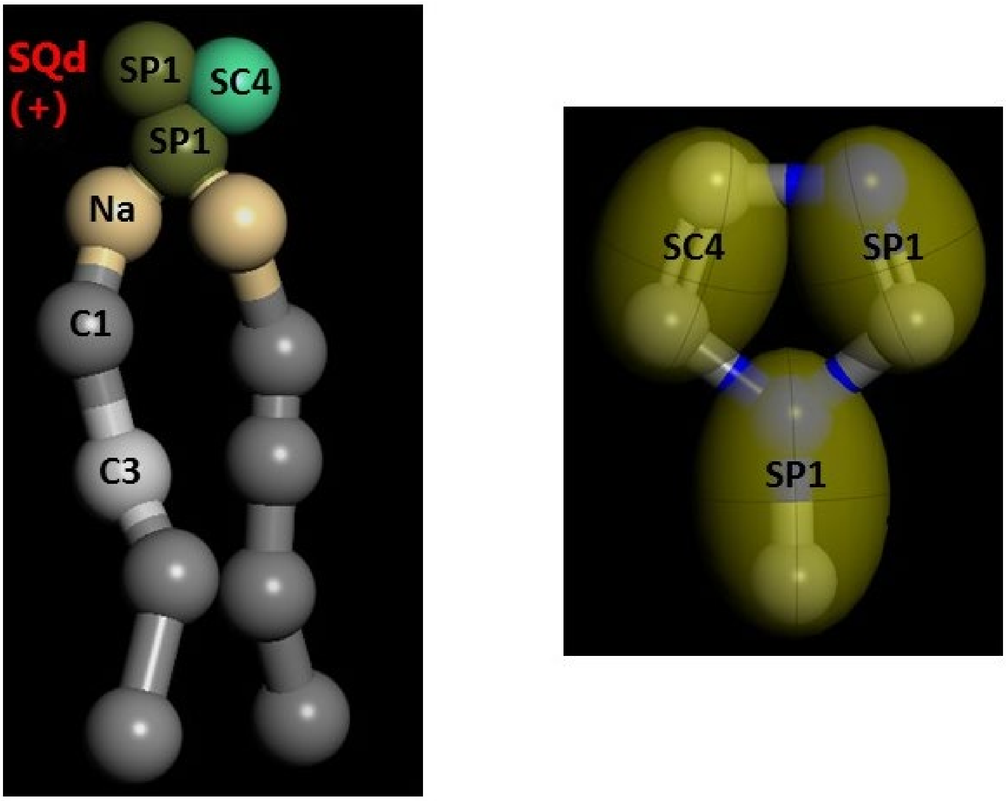

Figure S1. Coarse-grained model of ISUCA-Pal Ol (left) and imidazole ring showing bead types (right).

To parameterize the bonded interactions between imizadole ring (protonated and unprotonated) and glycerol, we used structural data that were obtained from comparison to all-atom simulations. The parameters for this new structure had to be calculated, as they do not exist in the literature. The all-atom simulations were converted into a "mapped" CG simulation by identifying the center-of-mass of the corresponding atoms as the CG bead. Some distribution functions were then calculated for the mapped simulation and compared to those obtained from a true CG simulation. Subsequently, the CG parameters were systematically changed until satisfactory overlap of the distribution functions were obtained.

The atomistic model of the lipid bilayer used to parameterize the coarse-grained model consisted of 32 ISUCA-2 Pal Pal lipids and 1600 water molecules (Figure S2).

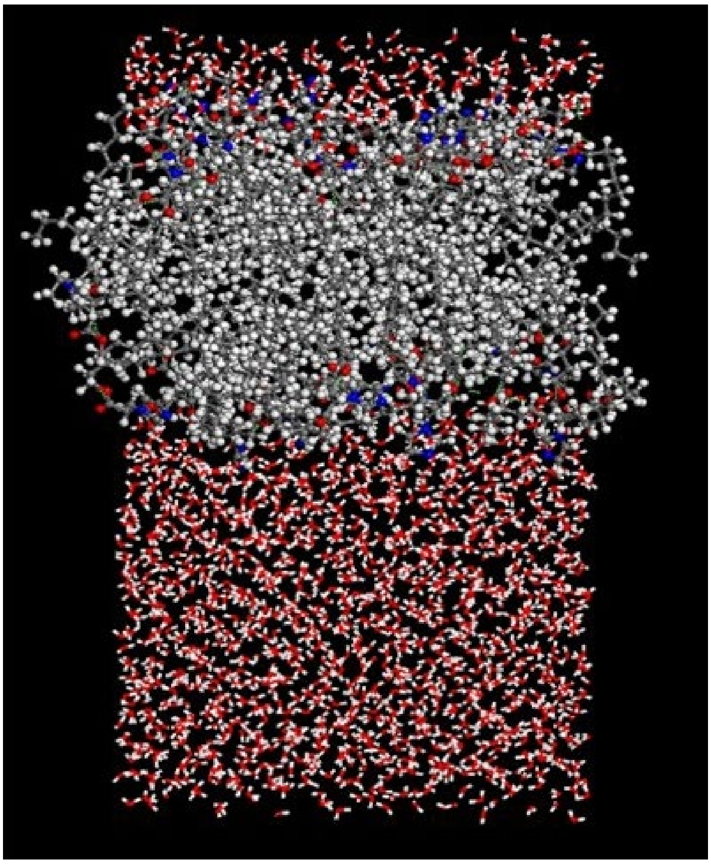

Figure S2. Atomistic model of a ISUCA-Pal Pal lipid bilayer.

To obtain the structural parameters needed to parameterize bonded interactions, we did a molecular dynamics simulation in the NPT ensemble during 2 ns using the COMPASS forcefield [5].

All-atoms and CG calculations results of some distances and angle distribution functions, as well as the radius of gyration of the lipid bilayer, are compared in Figure S3. The CG data were obtained using the bonded parameters shown in Table 2. Parameters not shown in this table were taken from references [6] and [7]. As can be seen, the lower resolution of the CG model does not allow a perfect reproduction of all the degrees of freedom of the underlying atomistic model. This is a well-known result previously found by other authors [S4, S5]. The parameters in Table S1 were finally chosen to run our CG molecular dynamics simulations.

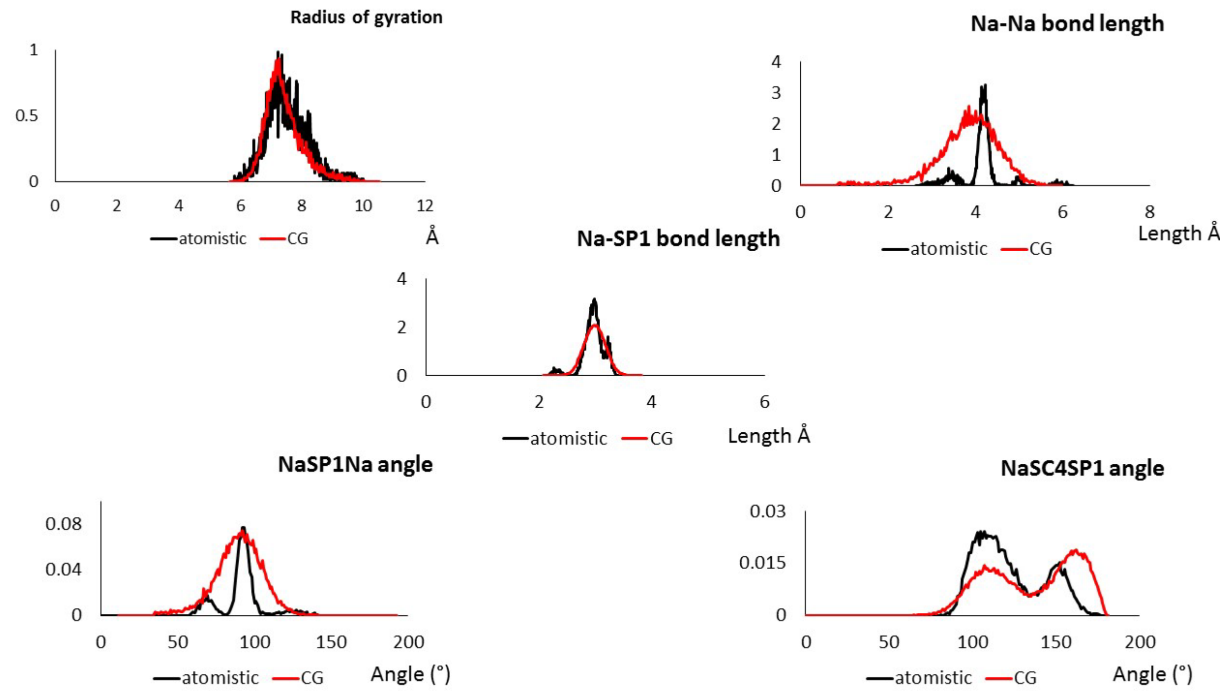

Figure S3. Comparison of structural parameters obtained from both atomistic and CG molecular dynamics simulations.

Table S1. Functional forms and parameters used to describe bonded interactions of the headgroup of ISUCA-derived lipids.

**Bonded Interaction**	**Functional form**	**Beads**	**Parameters**	
			**ko[1] (kcal/mol Å^2)**	**ro (Å)**
Bond stretch	$E = \frac{ko}{2}(r - ro)^2$	Na-Na	2.99	3.7
		Na-SP1	17.95	3.1
			**ko[1] (kcal/mol)**	**θo (°)**
Angle bending	$E = \frac{ko}{2}(\theta - \theta o)^2$	NaSP1SC4	12	115
		NaSP1Na	12	100
		NaSP1SP1	12	115
		NaSP1SQd	12	115

[1]Bond and angle force constants

References

1. Hollingsworth, S.A; Dror, RO. Molecular Dynamics Simulation for All. *Neuron*. **2018**, 99(6), 1129-1143. https://doi.org/10.1016/j.neuron.2018.08.011.

2. Scholarly Community Encyclopedia. https://encyclopedia.pub/entry/33716 (accessed on 9th February 2023)

3. Marrink, S.J.; Risselada, H.J. ; Yefimov, S.; Tieleman, D.P. ; de Vries, A.H. The MARTINI force field: coarse grained model for biomolecular simulations. *J. Phys. Chem. B* **2007**, 111, 7812–7824. https://doi.org/10.1021/jp071097f.

4. Djurre, H.; de Jong, G.S.; Bennett, W.F. ; Arnarez, C.; Wassenaar, T.A.; Lars, V.S.; Periole, X.; Tieleman, D.P.; Marrink, S.J.;Improved Parameters for the Martini Coarse-Grained Protein Force Field. *J. Chem. Theory Comput*. **2013**, 9,, 687–697. https://doi.org/10.1021/ct300646g.

5. Sun, H.; Pengyu, R.; Fried, J.R. The COMPASS force field: Parameterization and validation for phosphazenes. *Comput. Theor. Polym. Sci.* **1998**, 8, 229–246. https://doi.org/10.1016/S1089-3156(98)00042-7.

6. Alessandri, R.; Uusitalo, J.J.; de Vries, A.H:, Havenith,R.W.A.; Marrink, S.J: Bulk Heterojunction Morphologies with Atomistic Resolution from Coarse-Grain Solvent Evaporation Simulations. *J. Am. Chem. Soc*. **2017**, 139, 3697–3705. https://doi.org/10.1021/jacs.6b11717.

7. Qiu, L.; Liu, J.; Alessandri, R.; Qiu, X.; Koopmans, M,; Havenith, R.W.A.; Marrink, S.J.; Chiechi, R.C.; Koster, L.J. A.; Hummelen, J.C. Enhancing doping efficiency by improving host-dopant miscibility for fullerene-based n-type thermoelectrics. *J. Mater. Chem. A*. **2017**, 5, 21234-21241. https://doi.org/10.1039/C7TA06609K.
